# Impact of myocardial hemorrhage on left ventricular function and remodeling in patients with reperfused acute myocardial infarction

**DOI:** 10.1186/1532-429X-11-S1-O30

**Published:** 2009-01-28

**Authors:** Javier Ganame, Giancarlo Messalli, Steven Dymarkowski, Frank E Rademakers, Walter Desmet, Frans Van de Werf, Jan Bogaert

**Affiliations:** University Hospitals Leuven, Leuven, Belgium

**Keywords:** Left Ventricular Ejection Fraction, Infarct Size, Left Ventricular Volume, Microvascular Obstruction, Cine Magnetic Resonance Imaging

## Background

Myocardial hemorrhage is a common complication following reperfusion of ST-segment-elevation acute myocardial infarction (MI). Although its presence is clearly related to infarct size, at present it is unknown whether post-reperfusion hemorrhage affects left ventricular (LV) remodeling. Magnetic resonance imaging (MRI) can be used to identify myocardial infarction, myocardial hemorrhage and microvascular obstruction (MVO), as well as measure LV volumes, function and mass.

## Methods and results

Ninety-eight patients (14 females, 84 males, mean age: 57.7 years) with MI reperfused with percutaneous coronary intervention (PCI) were studied in the first week and at 4 months after the event. T2-weighted MRI was used to differentiate between hemorrhagic (i.e., hypo-intense core) and non-hemorrhagic infarcts (i.e., hyper-intense core). MVO and infarct size were determined on contrast-enhanced MRI, while cine MRI was used to quantify LV volumes, mass and function. Twenty-four patients (25%) presented with a hemorrhagic MI. In the acute phase, presence of myocardial hemorrhage was related to larger LV end-diastolic and end-systolic volumes and infarct transmurality, lower LV ejection fraction as well as lower systolic wall thickening in the infarcted myocardium (all p-values < 0.001). Infarct size, size of area at risk and size of MVO were significantly larger in patients with hermorrhagic MI. At 4 months, a significant improvement in LV ejection fraction in patients with non-hemorrhagic MI was seen (baseline: 49.3 ± 7.9% vs. 4 months: 52.9 ± 8.1%; p < 0.01). LV ejection fraction did, however, not improve in patients with hemorrhagic MI (baseline: 42.8 ± 6.5% vs. 4 months: 41.9 ± 8.5%; p = 0.68). Multivariate analysis showed myocardial hemorrhage to be an independent predictor of adverse LV remodeling at 4 months (defined as an increase in LV end-systolic volume). This pattern was independent of initial infarct size (See Table 1).

**Table 1:** Results of multiple linear regression of left ventricular remodeling

Predictors of end point	95% CI	R^2^	F value	p value
Hemorrhagic MI	0.15–0.31	0.17	20.19	<0.001
Infarct size at baseline	20.8–27.7	0.16	18.11	0.001
Microvascular obstruction	5.0–9.3	0.12	13.13	0.001
Maximum troponin I	91.9–142.6	0.10	10.75	0.001
Size of area at risk	37.0–46.5	0.09	9.12	0.003
LV mass at baseline	118.6–131.4	0.02	2.31	0.132
Percent MI transmurality	78.6–87.2	0.03	3.39	0.068
Infarct location	0.37–0.58	<0.01	0.41	0.892
Time to PCI	241.1–305.6	<0.01	0.02	0.874

## Conclusion

Myocardial hemorrhage, the presence of which can easily be detected with T2-weighted MRI, is a frequent complication after successful myocardial reperfusion, and an independent predictor of adverse LV remodeling regardless of initial infarct size.

